# Senescence as a Driver of Smooth Muscle Cell Plasticity and Atherosclerosis: Mechanisms and Therapeutic Opportunities

**DOI:** 10.3390/cells15020114

**Published:** 2026-01-08

**Authors:** Lisa Steegen, Mandy O. J. Grootaert

**Affiliations:** 1 Pole of Endocrinology, Diabetes and Nutrition, Institut de Recherche Expérimentale et Clinique (IREC), UCLouvain, 1200 Brussels, Belgium; lisa.steegen@kuleuven.be; 2 Center for Molecular and Vascular Biology, Department of Cardiovascular Sciences, KU Leuven, 3000 Leuven, Belgium

**Keywords:** senescence, smooth muscle cells, smooth muscle cell plasticity, atherosclerosis

## Abstract

**Highlights:**

**What are the main findings?**

- Senescent SMCs promote plaque instability through their pro-inflammatory profile.
- Senescence promotes SMC dedifferentiation and phenotypic modulation into plaque-destabilizing phenotypes.
- Senescence prevents SMCs from returning to a contractile state.

**What is the implication of the main finding?**

- Anti-senescence therapy may mitigate atherosclerosis by targeting the plaque-destabilizing SMC phenotypes.

**Abstract:**

Cell senescence is increasingly recognized as a key driver of atherosclerosis progression. Senescent smooth muscle cells (SMCs) lose their proliferative capacity and adopt a pro-inflammatory profile, contributing to impaired vessel repair and weakening of the fibrous cap. Moreover, senescence promotes SMC dedifferentiation and phenotypic modulation into unfavorable phenotypes associated with plaque destabilization. In this review, we will discuss how cell senescence is induced in atherosclerotic plaques, how this influences SMC plasticity, and how this impacts plaque stability. We will also evaluate the potential of current and experimental anti-atherosclerotic drugs to target SMC senescence and/or SMC phenotypic modulation.

## 1. Introduction

Atherosclerosis is a chronic inflammatory disease of the arterial wall characterized by the accumulation of lipids, immune cells, and vascular smooth muscle cells (SMCs). The contribution of SMCs to plaque formation and progression has long been underestimated, but owing to lineage-tracing and single-cell studies, SMCs have now been recognized as the most abundant cell type (±40–70%) present in atherosclerotic plaque (reviewed in [1]). When SMCs invade atherosclerotic plaque from the vessel wall, they lose their contractile properties but display remarkable plasticity, adopting proliferative, migratory, synthetic, or macrophage-like phenotypes that influence plaque growth and stability (reviewed in [2,3]). Besides this phenotypic modulation, SMCs also undergo cellular senescence during plaque progression, which is a state of irreversible growth arrest and an important hallmark of organismal aging [4]. With age, the number of senescent cells in the body increases, likely due to increased damage and/or reduced immune-cell-mediated clearance of senescent cells, although other hallmarks (e.g., stem cell exhaustion, epigenetic alterations) also independently drive aging [5]. In atherosclerotic lesions, SMCs undergo senescence either due to proliferative exhaustion or exposure to stressors (e.g., oxidative stress), causing stress-induced premature senescence (SIPS) [6]. Despite their inability to proliferate, senescent SMCs accelerate atherogenesis by promoting inflammation and fibrous cap thinning. Indeed, senescent cells are highly metabolically active and secrete cytokines, chemokines, and matrix-remodeling enzymes (defined as the SASP—senescence-associated secretory phenotype) that promote recruitment of immune cells and/or degradation of the fibrous cap [7,8,9]. Moreover, there is increasing evidence that cellular senescence acts as a critical regulator of SMC plasticity. Cellular senescence promotes SMC dedifferentiation and phenotypic modulation towards myofibroblast-like and osteoblast-like states whilst preventing SMCs from returning to a contractile state [9,10]. Conversely, clearance of senescent cells or modulation of SASP factors attenuates plaque burden and improves stability in part by reducing inflammation and promoting the formation of an SMC-rich fibrous cap [8,9,11]. Thus, senescence is not merely a terminal fate of SMCs but a pivotal determinant of their phenotypic trajectory in atherosclerosis. Understanding how senescence regulates SMC plasticity provides novel insights into disease pathogenesis and highlights therapeutic opportunities for senolytic or senomorphic interventions in vascular aging and atherosclerotic cardiovascular disease (CVD).

## 2. Vascular Smooth Muscle Cell Senescence

### 2.1. Characteristics of Senescent SMCs

#### 2.1.1. Markers of Senescent SMCs

Senescent SMCs are characterized by an irreversible growth arrest, typically following repeated cell divisions that result in telomere shortening and uncapping [6]. This ‘replicative senescence (RS)’ prevents genomic instability and accumulation of chromosomal abnormalities by stopping the proliferation of cells that are too old or damaged, thereby acting as an important tumor suppression pathway [4,12]. In addition to RS, SMCs can undergo SIPS in response to intrinsic factors, such as mitochondrial or genotoxic stress, or extrinsic stimuli, including oxidative stress, high glucose, UV radiation, and oncogene activation [6]. Unlike RS, SIPS arises rapidly and independently of telomere shortening but equally activates the DNA damage response (DDR) [12]. Activation of DDR triggers a cascade of DNA damage kinases, including ataxia telangiectasia and Rad3-related (ATR) and ataxia telangiectasia mutated (ATM), which subsequently activate checkpoint kinase 1 (CHK1) and checkpoint kinase 2 (CHK2). These kinases phosphorylate and activate p53, which induces transcription of the *CDKN1A* gene, encoding the cyclin-dependent kinase (CDK) inhibitor p21. Activated p21 binds to specific CDKs, preventing their interaction with cyclins and thereby inducing cell cycle arrest and cellular senescence [12]. DNA damage also activates CDKN2A (p16^INK4a^), which inhibits CDK4/6, maintaining retinoblastoma protein (RB) in its active form and suppressing E2F-mediated transcription of genes involved in cell cycle progression, resulting in cell senescence [12]. Both RS and SIPS share characteristic phenotypes, including increased cell size, granularity (e.g., caused by the buildup of components like lysosomal material, protein–lipid aggregates), and elevated senescence-associated β-galactosidase (SAβG) activity [13], the latter reflecting enhanced lysosomal content and activity at a suboptimal, less acidic pH in senescent cells [14]. SAβG is often a reliable marker for in vitro and in vivo detection of senescence in SMCs [7,10,15,16] but may show false-positive staining in lysosome-rich cells (e.g., macrophages) or tissues [17,18]. Other common markers used for the detection of SMC senescence include p16, p21, and p53, proliferation markers (e.g., Ki67, EdU labeling), and DNA damage markers (e.g., gH2AX), but none alone are specific for senescence [19]. Also, changes in the levels of nuclear proteins such as lamin B1 and HMGB1 are used as markers of human SMC senescence in vitro [11,20], yet none of these are specific for senescence [19]. In vivo, p16 is frequently used due to its low expression in young tissues, although it may have some limitations: some senescent cells do not, or only transiently, express p16, while it can be expressed by non-senescent cells such as activated immune cells [11,17,19]. Hence, for the detection of SMC senescence in mouse or human tissue samples, we recommend using a combination of p16 (or p21) immunohistochemistry, SAβG staining, and colocalization with a SMC marker. The downside is that SAβG staining only works on cryosections, not paraffin. Other potential markers are the long non-coding RNAs PURPL and NEAT1, both p53 targets, that were found to be upregulated in replicative senescent human SMCs [21]. Transcriptomic profiling of human senescent SMCs has led to the identification of two new markers, transmembrane protein 178B (TMEM178B) and secreted frizzle-related protein 4 (SFRP4) [10]. Urokinase-type plasminogen activator receptor (uPAR) could be another important marker; it is a cell-surface protein upregulated in senescent cells [22], including senescent SMCs [23], and it can also be released, acting as a biomarker of senescence. The SASP, characterized by the secretion of a wide variety of pro-inflammatory molecules and growth factors [24], is another important feature of senescent SMCs (see Section 2.1.2) [7]. As many of these factors are secreted by other cell types, including immune cells, no individual SASP factor can definitively confirm a senescent state [12,18,25]. In conclusion, no single biomarker reliably identifies senescence, nor do senescent cells express all markers; instead, a combinatorial approach of different markers is recommended for accurate detection of SMC senescence [19,25].

#### 2.1.2. Secretory Phenotype of Senescent SMCs

A hallmark of SMC senescence is its capacity to modulate the surrounding microenvironment through the SASP. The composition of the SASP varies depending on the cell type and senescence inducer [25]. In SMCs, the SASP typically includes a combination of pro-inflammatory cytokines (e.g., IL-1α, IL-1β, and IL-6), chemokines (e.g., IL-8, MCP-1, and CCL-2), extracellular matrix proteases (e.g., MMP-3 and MMP-9), growth factors, and other signaling molecules such as growth differentiation factor 15 (GDF15) [24,26,27,28]. Importantly, the SASP can act in both autocrine and paracrine manners. Through IL-1α-driven autocrine signaling, senescent SMCs reinforce their own senescent state by secreting cytokines and growth inhibitors that act on receptors on the same cell [27]. In parallel, paracrine signaling allows senescent cells to induce inflammation and DNA damage in neighboring healthy cells, known as ‘paracrine’ or ‘bystander’ senescence [24,29]. In some cases, the SASP can also exert pro-mitogenic stimulation of neighboring cells via cytokines like IL-6 [30]. Moreover, via the secretion of chemokines (e.g., CCL2), the SASP can act on the immune system, leading to immune cell recruitment and subsequent clearance of senescent cells [31]. However, senescent cells may also avoid elimination by expressing high levels of the “don’t eat me signal” CD47 that impairs macrophage-mediated efferocytosis [32]. Overall, the propagation of senescence amplifies the SASP response and creates a chronic pro-inflammatory milieu, establishing a positive feedback loop that contributes to the progressive accumulation of senescent cells [24]. The idea that cell senescence can spread locally, or even at a systemic level, is alarming and suggests that even a small number of senescent cells can have deleterious effects on disease. The main regulators of the SASP include a sustained DDR and the activation of key signaling pathways such as p38 MAP kinase and cGAS-STING [12]. In most cases, these pathways converge on the activation of Nuclear Factor kappa-light-chain-enhancer of activated B-cells (NF-κB) and CCAAT/enhancer-binding protein-β (C/EBPβ) that control the transcription of key SASP components [12]. For example, senescent SMCs release cytoplasmic chromatin fragments outside the nucleus, which trigger the SASP through the cGAS-STING-NF-κB pathway [7]. In addition to soluble SASP factors, emerging evidence indicates that extracellular vesicles (EVs), such as exosomes or microvesicles, contribute to paracrine senescence [33,34]. EVs serve as essential vehicles of intercellular communication, carrying proteins, lipids, metabolites, and nucleic acids such as mRNA and miRNAs. Senescent SMCs have been shown to secrete a higher number of EVs compared to non-senescent cells, which may act pro-inflammatorily. For example, Gluchowska et al. showed that several proteins, including Serpin Family F Member 1 (SERPINF1) and Thrombospondin 1 (THBS1), are upregulated components of EVs derived from senescent SMCs [28]. Moreover, EVs released by senescent SMCs induce the secretion of pro-inflammatory cytokines in neighboring T cells and monocytes [28]. The mechanism underlying senescence-induced EV secretion remains poorly understood [33,35] but is at least partially mediated by p53 and one of its downstream targets, tumor suppressor-activated pathway 6 [36]. p53 is also implicated in the upregulation of RAB proteins, which are important for endosome maturation [37]. Moreover, dysfunctional lysosomes in senescent cells may drive elevated EV secretion, as modulating lysosomal pH altered EV release in both senescent and proliferating fibroblasts [38]. Altogether, senescent SMCs remodel their microenvironment through both soluble SASP factors and increased EV secretion, reinforcing paracrine senescence and chronic inflammation. Hence, for diagnostic or monitoring purposes, combined detection of circulating SASP proteins, EV components, and other factors released by senescent cells (e.g., uPAR) could be exploited to assess senescence in blood, yet these approaches might be less SMC-specific.

### 2.2. Cellular Regulators of SMC Senescence

#### 2.2.1. NAD^+^

Nicotinamide adenine dinucleotide (NAD^+^) is an important redox cofactor and signaling molecule that drives mitochondrial energy metabolism and serves as a substrate for NAD^+^-consuming enzymes such as sirtuins (see Section 2.2.2) [39]. With age, NAD^+^ levels decline in multiple tissues, and low levels of NAD^+^ lead to several hallmarks of aging, including mitochondrial dysfunction, DNA damage, and cell senescence [39]. Why NAD^+^ levels decline with age is not completely uncovered, but the NADase enzyme CD38 is considered the major culprit [40,41], and cellular senescence may further propagate this [41,42]. Indeed, senescent cells increase CD38-NADase activity in non-senescent cells such as endothelial cells (ECs) or macrophages through their inflammatory SASP [41,42], suggesting a bidirectional link between senescence and age-related cellular NAD^+^ decline. In the vasculature, CD38 deficiency attenuates angiotensin (Ang) II-induced vascular remodeling, hypertension, and SMC senescence by suppressing the release of senescence-promoting EVs [43]. Hence, NAD^+^-boosting therapies, either by the stimulation of NAD^+^ synthesis or the inhibition of its degradation (e.g., by CD38), have become an attractive approach to mitigate (age-related) vascular diseases (see Section 5.3.1). Furthermore, reduced activity of nicotinamide phosphoribosyltransferase (NAMPT), the rate-limiting enzyme for NAD^+^ salvage from nicotinamide, induces premature senescence in human SMCs, whereas overexpression of NAMPT increases cell lifespan [44]. In vivo, loss of NAMPT promotes DNA damage, SMC senescence and aortic dissection in AngII-treated mice [45]. Altogether, the self-reinforcing loop between NAD^+^ loss and the SASP places NAD^+^ at the intersection of aging and cellular senescence.

#### 2.2.2. Sirtuins

Sirtuins are a family of NAD^+^-consuming deacetylase enzymes known for their role in longevity. Indeed, sirtuin levels and/or sirtuin activity decline with age, further supporting the direct link between low NAD^+^ levels, low sirtuin activity, and cell senescence [46]. Exposure to hyperlipidemic agents (such as oxidized LDL or fatty acids) reduces the expression of Sirtuin 1 (SIRT1) and Sirtuin 6 (SIRT6) in SMCs in human and mouse atherosclerosis and in human aortic SMCs [15,47]. Both sirtuins are (largely) found in the nucleus, where they control chromatin remodeling and transcriptional silencing by deacetylation of histones. As such, they regulate many cellular processes, including DNA damage repair, apoptosis, senescence, inflammation, antioxidant defense, and cell metabolism. SIRT6 has been shown to regulate telomere maintenance in human SMCs by deacetylating histone H3 lysine 9 (H3K9), thereby preventing telomeric DNA damage and cellular senescence [15]. In vivo, SMC-specific SIRT6 overexpression reduces atherosclerosis and cell senescence in mice, whereas expression of a SIRT6-inactive mutant increases several features of plaque instability [15]. In contrast to SIRT6, SIRT1 has many other non-histone targets such as transcription factors and coregulators, including key components of the inflammatory signaling pathway. For example, SIRT1 directly interacts and inhibits NF-κB through deacetylation of the p65 subunit. In a rat model of coronary artery spasms, SIRT1-mediated deacetylation of NF-κB alleviates the disease by inhibiting the myosin light chain pathway and the expression of endothelin-1 [48], but whether SIRT1 can inhibit senescence-associated inflammation in SMCs is not clear yet. Moreover, SIRT1 extends culture lifespan of human SMCs but only when NAMPT activity is also enhanced [49]. SIRT1 also protects SMCs against apoptosis and DNA damage by deacetylating and activating the repair protein Nijmegen Breakage Syndrome-1 (NBS-1) but not p53 [47]. In vivo, SMC-specific expression of an inactive SIRT1 protein exacerbates atherosclerosis and plaque instability, characterized by increased DNA damage and SMC apoptosis [47]. Conversely, SMC-specific overexpression of active SIRT1 reduces neointima formation after carotid artery ligation injury in mice [50] and attenuates Ang II-induced vascular remodeling and hypertension which was associated with lower ROS production, inflammation, and collagen synthesis in the arterial wall [51].

#### 2.2.3. Oxidative Stress

Oxidative stress is considered one of the main drivers of cell senescence [52]. Firstly, reactive oxygen species (ROS) generate DNA damage by acting as a mutating agent, leading to activation of the DDR and stress-induced senescence if the DNA damage remains unrepaired. Indeed, H_2_O_2_-induced SIPS correlates with increased levels of double-strand breaks and ROS [53], yet chronic oxidative stress can also trigger replicative SMC senescence [54]. Moreover, a positive feedback loop between p21 and mitochondrial ROS (mtROS) is essential for maintaining a stable growth arrest and senescent phenotype [55]. Secondly, ROS may induce senescence in neighboring cells by acting as signaling molecules and promoting a pro-inflammatory NF-κB-dependent SASP [56]. Hence, both telomere-dependent and -independent DNA damage trigger mitochondrial dysfunction, leading to enhanced ROS signaling, creating a vicious circle [55]. Besides mitochondria, NADPH oxidases (NOX) are a major source of ROS in the vascular wall. For example, NOX1 mediates AngII-induced senescence in SMCs by facilitating mitochondrial dysfunction and enhancing mtROS production [57]. Interestingly, NOX1/2 inhibition decreases vascular ROS levels and atherosclerosis in young, but not aged (i.e., 16 months old), ApoE^−/−^ mice [58,59], while NOX4 suppression decreases mtROS levels and improves mitochondrial function of SMCs isolated from old mice [58]. The authors also found that increased NOX4 expression in aortic SMCs of aged human patients was associated with increased mtROS and atherosclerosis severity. They further demonstrate that increased activation of NOX4 induces a proinflammatory SMC phenotype [60]. In contrast, others reported *NOX4* to be downregulated upon SMC senescence, whereas inhibition of NOX4 activity or decreased NOX4 levels lead to a pro-inflammatory senescent phenotype in the absence of DNA damage [53]. Taken together, the regulation of H_2_O_2_ formation by NOX4 may be harmful or protective depending on the levels and source of ROS as well as the presence of other stressors.

Besides damage to DNA, ROS can also contribute to oxidative modifications to other cellular components such as lipids. Lipid peroxidation increases with age and may contribute to cellular senescence by enhancing ROS production and mitochondrial dysfunction [61]. Recent work from Sun et al. revealed a pro-senescent role for ferroptosis, a form of regulated cell death characterized by excessive accumulation of ferrous iron (Fe^2+^)-driven lethal lipid ROS. Pro-ferroptotic signaling promotes SMC senescence and arterial aging by accelerating vascular NAD^+^ loss and promoting the release of a pro-senescent secretome [62]. Overall, ROS and mitochondrial dysfunction are both important causes and consequences of cellular senescence by controlling stable growth arrest and a pro-inflammatory SASP.

#### 2.2.4. Autophagy

There is convincing evidence that autophagy, a cellular process by which the cell degrades and recycles cellular components for reuse, protects SMCs from senescence, thereby attenuating atherosclerosis [63,64,65,66]. Also in vitro, activation of autophagy using the mTOR inhibitor rapamycin attenuates oxLDL-induced [63] and doxorubicin-induced SMC senescence [67]. On the other hand, blocking autophagy through deletion of the autophagy gene *Atg7* induces premature senescence in mouse aortic SMCs and accelerates atherosclerosis in mice [64]. Autophagy-defective SMCs are also characterized by impaired mitochondrial quality control and increased oxidative stress [66], which may facilitate the senescent phenotype of these cells. Others also reported increased apoptosis of autophagy-deficient SMCs upon exposure to atherogenic lipids [65,66]. It is important to note that the autophagic activity declines with age and that autophagy becomes impaired or dysfunctional in advanced atherosclerosis [68]. Furthermore, loss of SMC autophagy exacerbates Ang II-associated aortic remodeling [69] and the development of dissecting aortic aneurysms, which were characterized by reduced SMC proliferation, increased SMC death, ER stress, and inflammation [70]. All together, these studies illustrate that autophagy may regulate different SMC fates (e.g., cell death, cell senescence) depending on the environmental context [71].

#### 2.2.5. SMC-Specific Transcriptional Regulators

Apart from p53 and SIRT1 and SIRT6 acting as epigenetic regulators of transcription, there are not many transcription factors known that regulate the senescence phenotype in SMCs [72]. However, loss of transcriptional regulators such as myocardin (MYOCD) and p300 that control the contractile SMC phenotype is associated with increased SMC senescence [10]. In contrast, SMC-specific loss of the transcription factor Kruppel-like factor 4 (KLF4), a master regulator of SMC phenotypic modulation [73], leads to reduced plaque senescence [74]. KLF4 also inhibits SMC proliferation by direct activation of the *p21* gene acting in concert with p53 [75]. As such, KLF4 could act as a critical switch mediating both SMC phenotypic modulation and senescence. However, there is another report showing that KLF4 overexpression prevents SMC senescence by activating autophagy [76]. At the moment, there is no strong evidence that SMC-specific transcriptional regulators such as MYOCD directly regulate senescence genes, but loss leads to SMC dedifferentiation, which may predispose the cell to senescence.

### 2.3. Evidence of SMC Senescence in Atherosclerosis

Within atherosclerotic plaques, SMCs are exposed to oxidative stress, inflammatory mediators, modified lipids, and repeated proliferative cycles, all known inducers of cellular senescence (reviewed in [71]). As such, senescence is considered a downstream response to atherogenic stressors. However, there is a significant amount of evidence that SMC senescence promotes the progression of atherosclerosis and destabilizes the fibrous cap by actively altering plaque composition [77,78]. Hence, senescence is both a consequence of atherosclerosis and an upstream driver that amplifies inflammation and destabilizes plaques. The number of senescent SMCs in an atherosclerotic plaque is not fixed but depends on the size of the plaque, the vascular bed, and the individual and generally increases with disease stage. The identification of senescent cells in the plaque, however, is being challenged by the lack of a universal senescence marker. Often, SAβG is solely used as marker, but it should be noted that plaque macrophages, and thus potentially also macrophage-like SMCs, may show false-positive staining due to their high lysosomal content. The quantification of senescent cells in human plaques is further challenged by the fact that most available tissues, either from carotid endarterectomies or post-mortem (coronary) artery samples, are late-stage plaques. Because senescent cells accumulate slowly, it is difficult to pinpoint a defined onset of senescence in human atherosclerosis. Moreover, human lesions typically develop over decades influenced by lifelong exposure to risk factors and chronological aging itself, challenging the quantification of senescent cell burden in human plaques. In mice, however, we can control lesion progression by adjusting the duration of the high-fat diet, making it easier to map when senescence markers appear relative to plaque initiation and progression. Also, genetic-lineage-tracing tools make it possible to identify SMC-derived senescent cells in the plaque at specific time intervals. One limitation might be that the duration of the disease is ‘compressed’, and even aged mice (e.g., 24 months old) do not fully recapitulate chronological aging and senescence that accrues over many years in humans.

#### 2.3.1. SMC Senescence in Mouse Atherosclerotic Plaques

In mice, approx. 2–20% of plaque SMCs display senescence markers at advanced stages [8,9,15]. Moreover, telomere-associated DNA damage foci have been observed in 45% of all medial SMCs in fat-fed ApoE^−/−^ mice [79]. Accumulation of senescent, and thus non-dividing, SMCs paradoxically promotes plaque growth, at least in part by promoting inflammation [7]. The implementation of senolytic therapy has facilitated the investigation of the role of cellular senescence in atherosclerosis, revealing that the selective removal of senescent cells can reduce plaque burden and promote features of plaque stability. For example, the senolytic drug ABT-263 (aka., Navitoclax) reduces plaque size [8,11], improves plaque stability (e.g., thicker fibrous caps, more collagen deposition), and lowers (plaque) inflammation in mice [8]. Instead, the disease outcomes of genetic approaches to remove p16+ cells are conflicting [8,11].

One way forward to study the role of senescent SMCs is by analyzing their transcriptome. Bennett’s group applied single-cell RNA sequencing (scRNAseq) on mouse atherosclerotic plaques to identify new SMC senescence markers. They revealed that senescent SMCs, both in vitro and in vivo, are characterized by expression of TMEM178B and SFRP4 and are associated with a dedifferentiated phenotype [10]. Future “omics” studies designed to assess the transcriptomic signature of senescent plaque SMCs in the presence/absence of senolytics will further improve our understanding on the role of SMC senescence in atherosclerosis.

#### 2.3.2. SMC Senescence in Human Atherosclerosis

Senescent SMCs can make up a substantial fraction of SMCs in advanced human plaques, with estimates ranging from 5–20% of SMCs showing markers of senescence (e.g., p16, p21, SAβG). Indeed, immunohistochemical staining of advanced human lesions shows colocalization of p16 and p21 with SAβG-positive SMCs, while these markers were absent in normal healthy vessels [54]. p53 expression was increased in whole lysates of human plaques compared with normal vessels and increased in SMCs in plaques compared with normal aorta [80]. SMCs derived from human plaques are also characterized by higher expression levels of p16 and p21, hypophosphorylation of RB, a large, flattened cell shape, and SAβG activity when compared to normal aortic SMCs [47]. Besides the classical SIPS markers, advanced human plaques also show evidence of telomere-based replicative senescence [54]. Indeed, telomeres in fibrous cap SMCs are significantly shorter compared with medial SMCs, and telomere shortening is positively correlated with disease severity [54]. Human plaque-derived SMCs also exhibit reduced expression and binding of the telomeric protein TRF2 to telomeres and increased DNA damage and DDR activation at the telomeres, all correlating with senescence [80]. Hence, we propose that replicative senescence is attributed to enhanced clonal proliferation of plaque SMCs [81], although it may also be accelerated by oxidative-stress-induced DNA damage [54].

## 3. Vascular Smooth Muscle Cell Plasticity

SMC lineage-tracing and scRNAseq studies have revealed a remarkable diversity and plasticity of plaque SMCs in mice and humans (reviewed in [1,2,3]). Some of these SMC phenotypes adopt plaque-stabilizing fates by forming a protective fibrous cap, while others transition into phenotypes that may impede plaque stability, as discussed below.

### 3.1. Contractile vs. Synthetic

Originally, SMC phenotypic switching was described as the switch from a contractile to a synthetic phenotype defined by the loss of contractile markers (e.g., ACTA2, MYH11, TAGLN, and calponin), production of synthetic markers (e.g., extracellular matrix proteins, vimentin), and increased migratory and proliferative capacity [1,2,3]. Importantly, 80% of SMC-derived cells in advanced atherosclerotic plaques are undetectable by histology for typical SMC markers (e.g., ACTA2) because of the downregulation of these contractile markers [82]. SMC phenotypic switching occurs in response to environmental cues such as inflammation, lipid accumulation, and mechanical stress and plays a crucial role in intimal thickening and plaque formation [3]. Although the majority of studies focus on phenotypically switched SMCs inside the plaque, it is important to highlight that loss of contractile markers already occurs in the medial layer underneath the plaque [83], likely playing a primary role in SMC invasion of the plaque. Fibrous-cap SMCs may also (re-)express contractile proteins to some extent, but compared to the medial SMCs, they have acquired a more synthetic phenotype to form a protective fibrous cap [84]. It is important to add that about 40% of ACTA2^+^ cells in the fibrous cap are derived from non-SMCs, including ECs that have undergone endothelial-to-mesenchymal transition (EndoMT) and macrophages undergoing macrophage-to-myofibroblast transition [85].

### 3.2. Macrophage-like and Foam-Cell-like

The original observations that SMCs can transdifferentiate into macrophage-like cells upon cholesterol loading in vitro have been corroborated by recent scRNAseq experiments identifying clusters of macrophage-like and foam-cell-like SMCs in mouse [86] and human [87] plaques. These types of cells have macrophage-like properties such as phagocytic and efferocytotic activity (albeit at a lower efficiency) and lipid uptake and express typical macrophage markers such as CD68, LGALS3 (Galectin-3), and Mac-2 (reviewed in [1,2,3]). Interestingly, SMC lineage-tracing studies revealed that the majority of foam cells (~between 50–70% of all plaque cells) are in fact SMC-derived [86,87] and have thus been previously falsely marked as macrophages. However, whether macrophage-like SMCs behave as ‘true’ macrophages is still under debate, as their transcriptional profile is very different from classical macrophages and they have a poorer phagocytic capacity [88]. Nevertheless, they are thought to promote plaque progression and instability by promoting lipid accumulation and inflammation.

### 3.3. Myofibroblast-like

Myofibroblast-like (or fibromyocyte) SMCs exhibit a fibroblast-like transcriptional profile characterized by expression of TNFRSF11B (aka., osteoprotegerin), fibronectin 1, fibromodulin, collagen 1a1, and proteoglycans such as lumican [1,2,3]. Myofibroblast-like cells have been identified in scRNAseq datasets of mouse and human plaques [10,73,89], and they mostly accumulate in the fibrous cap and are considered to play a protective role in atherosclerosis. Their transition is, at least partially, regulated by the transcription factor TCF21, as SMC-specific loss of TCF21 reduces the number of myofibroblast-like cells in the cap and impairs plaque stability [89].

### 3.4. Osteogenic- and Chondrogenic-like

In calcified regions of the plaque, SMCs can transdifferentiate into osteogenic- and chondrogenic-like cells. Osteogenic-like SMCs are characterized by upregulation of runt-related transcription factor 2 (RUNX2), osteopontin, bone morphogenetic protein 2 (BMP2), and alkaline phosphatase activity, propagating calcification. Chondrogenic-like SMCs express markers such as SRY-related HMG box 9 (SOX9) and produce a cartilage-like matrix [1,2,3]. According to lineage-tracing studies, 98% of all osteochondrogenic cells in plaques are of SMC origin [90]. Apoptotic bodies derived from SMCs also facilitate vascular calcification [91]. The role of plaque calcification is rather complex, but it is generally accepted that microcalcifications are detrimental for plaque stability, as these are tiny, fine calcium deposits often found in the fibrous cap, while macrocalcifications may stabilize the plaque, as they are found in organized structures typically deeper within the plaque that are more resistant to mechanical stress [92].

## 4. Plaque-Destabilizing Effects of Senescent SMCs Through Modulation of SMC Plasticity

There is growing evidence that the plaque-destabilizing effects of senescent SMCs go beyond inflammation (see Section 4.4). Senescence also promotes plaque instability by inducing fibrous cap thinning (see Section 4.1), SMC dedifferentiation (see Section 4.2), and SMC calcification (see Section 4.3).

**Figure 1:**
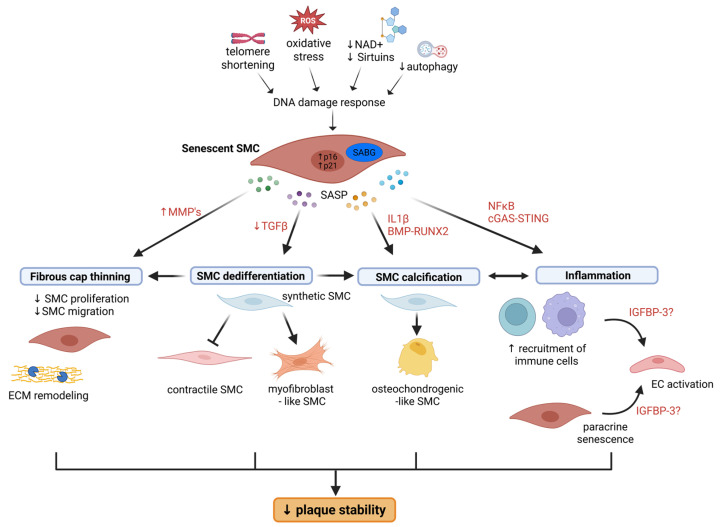
Schematic illustration of how SMC senescence contributes to atherosclerotic plaque instability. Senescence promotes (1) fibrous cap thinning by inhibiting SMC proliferation and migration and by promoting ECM remodeling through increased activity of MMPs; (2) SMC dedifferentiation into myofibroblast-like cells and preventing redifferentiation to a contractile state due to downregulated TGFβ signaling; (3) transdifferentiation into osteochondrogenic-like SMCs and vascular calcification through IL1β- and BMP-RUNX2-mediated signaling; and (4) vascular inflammation leading to EC activation and recruitment of immune cells to the lesion. While inflammatory signals can drive transformation into osteochondrogenic-like SMCs, SMC calcification, and in particular microcalcifications, become a source of further inflammation, The SASP factor IGFBP-3 may play a role in paracrine senescence. BMP, bone morphogenetic protein; ECM, extracellular matrix; IGFBP-3, insulin growth factor binding protein 3; IL1β, interleukin 1 beta; MMP, matrix metalloproteinases; NAD, nicotinamide adenine dinucleotide; NF-κB, nuclear factor-kappa B, RUNX2, runt-related transcription factor 2; SASP, senescence-associated secretory phenotype; TGFβ, transforming growth factor beta.

The idea that senescence could affect SMC plasticity arises from earlier observations where murine atherosclerotic plaques with SMC senescence showed thinner fibrous caps, reduced number of ACTA2+ cells and reduced/altered extracellular matrix deposition [10,15,80]. The direct link between cell senescence and plaque instability (Figure 1) is further corroborated by studies using senolytic approaches, which reduces necrotic core size [11] and enhances fibrous cap thickness and/or SMC content [9].

### 4.1. Thinner Fibrous Caps

The thickness of the fibrous cap is a key marker for plaque vulnerability. Work from Childs et al. showed that treatment of atherosclerotic mice with the senolytic drug ABT-263 led to thicker fibrous caps characterized by an increased numbers of SMCs with a matrix-producing phenotype [9]. Using SMC lineage-tracing techniques, the authors further showed that senolytic therapy enhances the number of vimentin+ SMC-derived cells—which are considered to be promigratory—in the SMC layer of the media underneath and closest to the plaque (which are called the first interfiber space) [9]. These cells are thought to cross the interfiber space and invade the plaque through breaks in internal elastic lamina, eventually reaching the fibrous cap. Overall, the authors put a mechanism forward by which cell senescence suppresses migration of medial SMCs, thereby preventing their accumulation in the fibrous cap. However, knowing the identity of these senescent cells would provide further insights into the pathological role of cell senescence in atherosclerosis. In contrast, Owen’s group found that ABT-263 treatment reduces SMC content by 90% and reduces ACTA2+ fibrous cap thickness by 60% in advanced plaques [74]. Mechanistically, ABT-263 treatment induced apoptosis of both senescent and non-senescent SMCs and ECs and prevented investment of endothelial-cell-derived cells into the fibrous cap via EndoMT. ABT-263 treatment, even at lower doses, led to a 50% mortality rate [74]. The discrepancies between both studies may be due to differences in the experimental design (number of weeks of HFD, HFD followed by low-fat diet, ApoE^−/−^ vs. LDLR^−/−^ mice), yet the senolytic protocol (100 mg/kg i.p.) was identical. Nevertheless, these studies raise the possibility that some effects of ABT-263 are not through senolysis and urge the need for developing novel senolytic agents that are less toxic.

Senescent SMCs may also drive plaque vulnerability through reduced collagen deposition and increased production of matrix-degrading enzymes (MMPs) (Figure 1). Senescent human SMCs produce less collagen and more matrix metalloproteinase-9 (MMP-9) compared to normal SMCs [27], but whether this is true in vivo is less clear and likely more complex. Indeed, scRNAseq of plaque-derived SMCs revealed higher expression of dedifferentiation markers and differential regulation of pathways associated with extracellular matrix organization in the senescent SMC population [10] (see Section 4.2).

### 4.2. SMC Dedifferentiation

While the work from Childs et al. revealed how senolytic therapy impacts SMC fate [9], Bennett’s group further focused on the autonomous effects of SMC senescence on cell plasticity [10] (Figure 1). The authors revealed that senescent SMCs acquire a dedifferentiated/fibromyocyte phenotype characterized by the expression of TNFRSF11B and fibromodulin as well as the newly identified ‘SMC senescence markers’ TMEM178B and SFRP4 [10]. ScRNAseq of lineage-traced SMCs in mouse and human plaques showed that p16+ SMCs had lower contractile marker expression and higher expression of dedifferentiated SMC markers (e.g., lumican, elastin, decorin, collagen 1a1, collagen 3a1) [10]. Similar results were obtained in mice expressing a SMC-restricted mutant TRF2 protein (TRF2T188A) that induces premature senescence, showing differential regulation of pathways associated with extracellular matrix organization, inflammation, and transforming growth factor-β (TGF-β). [10]. Moreover, the authors found that senescent SMCs were more resistant to re-differentiation towards a contractile phenotype due to a dysregulated TGF-β signaling. This was due to activation of the STING–TBK1–IRF3 pathway in response to accumulation of cytosolic DNA in senescent SMCs, as silencing of IRF3 restored expression of TGF-β pathway members and SMC contractile markers [10]. Although the myofibroblast phenotype is usually considered to be beneficial for plaque stability, the authors postulate that SMC senescence may promote plaque instability by impeding the cells from returning to a contractile state. Indeed, plaques of mice with accumulating senescent SMCs show a reduced number of ACTA2+ cells in the fibrous cap yet no difference in cap area [10], suggesting that either ACTA2-SMC or non-SMC-derived cells are occupying the cap, which could result in reduced stability. The plaques of these mice were also larger and had an increased necrotic core, suggesting increased plaque vulnerability. Furthermore, although these SMCs exhibited a pro-inflammatory signature [10] and SMCs can transform into macrophage-like cells, it is unclear at this point whether SMC senescence also promotes transition into a macrophage-like phenotype, which could further impede plaque stability.

### 4.3. SMC Calcification

There are several lines of evidence that SMC senescence promotes vascular calcification (Figure 1). For example, human SMCs undergoing replicative senescence in culture show increased expression of alkaline phosphatase and collagen 1a1, suggesting an osteoblastic transition, which was mediated by RUNX-2 [93]. Similarly, interleukin-1β-induced senescence promotes SMC osteoblastic transition in a BMP2-dependent manner in vitro [94]. Moreover, in a klotho-deficient mouse model of aging, vascular senescence was associated with medial calcification and osteoblastic transition of SMCs involving BMP2-RUNX2 signaling [95]. Work from Childs et al. further suggests that SMC senescence promotes calcification in atherosclerotic plaques, as senolytic treatment with ABT-263 reduced plaque calcification associated with the accumulation of RUNX-1+ SMC-derived cells in the plaque core and downregulation of multiple pro-calcification genes [9], although it is unclear through which mechanisms. Despite the differential effects of micro- vs. macrocalcifications on plaque stability, the authors did not specify this (i.e., alizarin red staining detects both types of calcification but was mostly seen in the core), and so it is unclear how this would then translate into plaque stability. We speculate that SMC calcification contributes to fibrous cap thinning, mainly indirectly via SMC phenotypic modulation and SMC death or directly when microcalcifications form in the cap, causing local stress and increased risk of rupture.

### 4.4. Inflammation

It is generally accepted that senescent cells, including senescent SMCs, promote plaque instability, at least partially through their pro-inflammatory SASP (Figure 1). Indeed, senescent SMCs may actively contribute to the chronic inflammation associated with atherosclerosis through a IL1α-driven SASP [27]. Besides promoting chemotaxis of macrophages, senescent human SMCs also prime neighboring healthy endothelial cells and SMCs to a proinflammatory state in vitro [27], underlining the paracrine effects of SMC senescence. These findings are corroborated by more recent and in vivo evidence that SMC senescence promotes EC activation and immune cell recruitment (e.g., macrophages, T cells) to the neointima through their SASP [7]. Activation of the cGAS-STING pathway led to an NF-κB-dependent pro-inflammatory SASP consisting of several interleukins and chemokines [7]. Although these studies advocate for the role of senescent SMC paracrine signaling in promoting disease progression, it is hard to study this in vivo.

Childs et al. further focused on one particular SASP factor, insulin growth factor binding protein 3 (IGFBP-3) [9], which is a potent inhibitor of insulin growth factor 1 (IGF1). Treatment with ABT-263 reduces IGFBP-3 expression in plaques, both in SAβG+ and SAβG- cells, which may potentially indicate that senescent cells induce IGFBP-3 expression in neighboring non-senescent cells through paracrine signaling [9]. Interestingly, anti-IGF1 treatment increases the number of vimentin+ACTA2+ cells in human plaque explants and in atherosclerotic mice, suggesting that an IGF1-dependent SASP may limit medial SMC migration and entry in the plaque [9]. However, an effect on fibrous cap thickness was not revealed, nor did the plaque size or senescence burden change upon anti-IGF1 treatment. Hence, it remains speculative whether or not anti-IGF1 therapy would improve plaque stability and whether this is through anti-senescence effects. Nevertheless, senolytic treatment with ABT-263 reduces fibrous cap thickness and IGFBP-3 expression levels [9], implying that targeting one particular SASP factor might be not sufficient to improve disease outcome. Indeed, other SASP factors undoubtedly also modulate SMCs in atherosclerosis, and so their identification as well as knowing the identity of these SASP-producing cells would give further insights into future therapeutic approaches.

## 5. Anti-Atherosclerotic Drugs That Inhibit SMC Senescence and/or SMC Dedifferentiation

**Figure 2:**
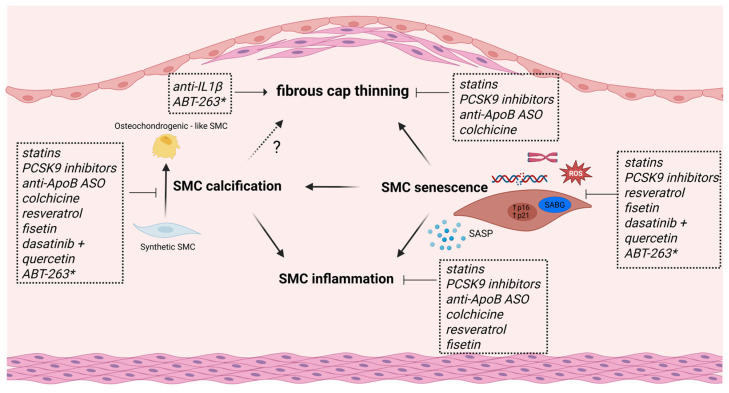
Schematic overview of current and experimental anti-atherosclerotic drugs that target SMC dedifferentiation and/or SMC senescence. Statins and PCSK9 inhibitors promote fibrous cap thickness, reduce vascular inflammation and calcification in vitro, and inhibit SMC senescence through various actions. Colchicine and ApoB-mRNA-targeting antisense oligonucleotides (ASOs) both reduce SMC inflammation and calcification while promoting fibrous cap stability, but whether they can also inhibit senescence is not known. Despite its beneficial therapeutic effects in human trials, anti-IL1β treatment leads to reduced SMC content and fibrous cap thinning in mice. The flavonoids resveratrol and fisetin reduce SMC inflammation and calcification in vitro and inhibit SMC senescence through various mechanisms. The senolytic cocktail dasatinib + quercetin exerts anti-atherosclerotic effects, in part by inhibiting SMC calcification and SMC senescence. The potential of the senolytic drug ABT-263 as an anti-atherosclerotic drug is less clear (*); while most research shows increased plaque stability, one study reported fibrous cap thinning and SMC loss in advanced lesions.

Several studies hint that some of the therapeutic effects of both current and novel anti-atherosclerotic drugs may be due to changes in SMC phenotype. Moreover, evidence from pre-clinical studies suggests that these drugs, including some experimental drugs, inhibit SMC senescence (Figure 2).

### 5.1. Established Anti-Atherosclerotic Therapies

Current atherosclerotic treatment focuses on risk factor management by combining lifestyle changes and lipid-lowering therapy such as statins and proprotein convertase subtilisin-kexin type 9 (PCSK9) inhibitors.

#### 5.1.1. Statins

Cholesterol-lowering statins reduce cardiovascular morbidity and mortality by ~20% and increase fibrous cap thickness in patients [96] but are also known for their pleiotropic effects (e.g., reduction in EC dysfunction, oxidative stress, and inflammation) that may enhance plaque stability [97]. Moreover, atorvastatin reduces SMC senescence and telomere shortening in vitro [98]. These effects were not mediated by changes in telomerase expression/activity or expression of telomere-associated proteins; instead, atorvastatin accelerated NBS-1-dependent DNA damage repair [98]. Simvastatin reduces SASP and ROS production and improves mitochondrial respiration in doxorubicin-induced and replicative senescent SMCs in an HMG-CoA reductase-dependent manner [16]. Fluvastatin was found to reduce SMC calcification by inhibiting P(i)-induced apoptosis in replicative senescent SMCs [93]. Some studies propose that these lipophilic statins may act, either as senolytic agents, as they induce senescent EC death at high doses [99], or as senomorphic agents by antagonizing the inflammatory SASP in senescent fibroblasts [100]. Along these lines, cholesterol lowering, either through switching to a low-fat diet or using ApoB-mRNA-targeting antisense oligonucleotides, depletes mouse atherosclerotic lesions of SMC-derived fibromyocytes and chondromyocytes in an NF-κB-dependent manner while leaving the population of stabilizing cap SMCs intact [101]. Although it still needs to be seen if these alterations in SMCs also occur in human atherosclerosis, these studies may indicate that lipid-lowering (drugs) may reduce atherosclerosis by modulating SMC function, phenotype, and senescence.

#### 5.1.2. PCSK9 Inhibitors

The anti-PCSK9 drugs (e.g., evolocumab) are a newer class of cholesterol-lowering drugs that successfully reduce cardiovascular events including cardiovascular mortality, even in patients on statin therapy [102]. Evidence is emerging that these drugs reduce atherosclerosis and fibrous cap thinning also through mechanisms independent of their cholesterol-lowering effect, particularly by altering SMC phenotype. Indeed, besides its expression in the liver, PCSK9 is also expressed in atherosclerotic plaques, with markedly higher expression levels in SMCs compared to other cell types [103]. There is some in vitro evidence that PCSK9 stimulates the differentiation of SMCs from a contractile to a synthetic phenotype [104] and may also induce SMC senescence [105] and SMC calcification [106]. Treatment with evolocumab reduced neointima formation in mice, which was phenocopied in PCSK9-knockout mice [107]. It is also plausible that PCSK9 inhibitors mediate senescence-associated inflammation, as PCSK9 deletion inhibits inflammasome activation in vascular SMCs [108]. Further investigation of the precise molecular mechanisms by which PCSK9 modulates SMC biology and atherosclerosis are warranted to determine the effect of PCSK9 inhibitors on SMC phenotype and senescence.

### 5.2. Emerging Anti-Atherosclerotic Drugs

The therapeutic focus is shifting from managing risk factors to directly targeting the disease process, particularly inflammatory pathways such as IL1β.

#### 5.2.1. Anti-IL1β Therapy

Anti-IL1β therapy has been shown to be beneficial in atherosclerotic patients by inhibiting inflammation, as demonstrated by the CANTOS trial [109], but its role on plaque stability is complex because IL1β has both detrimental and protective effects on SMCs. While IL1β generally promotes SMC transition to a pro-inflammatory state [110], SMC proliferation and migration [111], and even SMC senescence [94], it may also promote the formation and maintenance of a protective SMC-rich fibrous cap in advanced atherosclerosis [112]. Indeed, Gomez et al. found that anti-IL1β treatment of SMC-lineage-traced ApoE^−/−^ mice leads to reduced SMC and collagen content in advanced plaques due to reduced SMC proliferation [112]. Similarly, SMC-specific loss of IL1 receptor 1 results in plaques with thinner fibrous caps devoid of SMCs [112]. Therefore, targeting IL1β in SMCs requires careful consideration of the disease stage to avoid potential harm.

#### 5.2.2. Colchicine

Colchicine is an old anti-inflammatory drug that has been successfully repurposed for the secondary prevention of atherosclerotic CVD. Recent clinical trials have demonstrated that a low dose of colchicine reduces major adverse cardiovascular events in atherosclerotic patients, including those with an acute coronary syndrome, by ~30% [113]. Studies in mice showed that colchicine reduces early atherosclerosis by reducing leucocyte recruitment and vascular inflammation [114]. Colchicine also promotes stability of advanced plaques by promoting SMC modulation into protective myofibroblast-like cells through TGFβ and Notch 3 signaling, resulting in thicker fibrous caps rich in ACTA2+ SMC-derived cells [115], which was not mediated through inflammatory pathways. Instead, the authors found a reduction in SMC-derived RUNX2+ osteoblast-like cells and reduced intraplaque calcification upon colchicine treatment. In contrast, others reported that colchicine inhibits vascular calcification in an inflammation-dependent manner [116]. Whether colchicine could also impact atherosclerosis and fibrous cap stability by influencing SMC senescence is not known, but it does prevent oxidative-stress-induced senescence in EC by blocking NF-κB and MAPK signaling pathways [117].

### 5.3. Experimental Anti-Senescence Compounds with Anti-Atherosclerotic Effects

Anti-senescence drugs can be subdivided into senomorphics (aka senostatics) and senolytics. Senomorphics are compounds that suppress harmful inflammation and SASP from senescent cells. They generally operate by blocking pathways like NF-κB, mTOR, and MAPK, thereby reducing chronic inflammation and senescent cell spreading. Senolytic compounds kill senescent cells by targeting the anti-apoptotic pathways (e.g., Bcl2) that are often upregulated in senescent cells [118]. Given the breadth of available compounds, we refer the reader to several excellent review articles that provide a comprehensive overview of their modes of action and performance in (pre)clinical studies [119,120,121,122]. Here, we aim to focus on senomorphic compounds that exert anti-senescence effects on SMCs by boosting cellular NAD^+^ or activating sirtuins (Section 5.3.1) and on the senolytic drugs that are currently being tested for clinical use in CVD (Section 5.3.2).

#### 5.3.1. Senomorphics

Boosting intracellular NAD^+^ levels emerges as a promising senotherapeutic strategy in CVD. Preclinical evidence shows that supplementation with the NAD^+^ precursor nicotinamide mononucleotide (NMN) alleviates Ang II-induced vascular remodeling and senescence [43]. Mechanistically, NMN reverses Ang II-induced SMCs senescence in vitro by downregulating *Klf4* and *p16* through AMPK activation [123]. Along these lines, treatment with the CD38 inhibitor 78c alleviates Ang II-induced vascular remodeling and SMC senescence in an NAD/sirtuin-dependent manner [43]. Resveratrol, a polyphenol mostly found in red grapes and a well-known sirtuin activator, acts as a senomorphic agent mostly by suppressing inflammation. Resveratrol reduces atherosclerosis in ApoE^−/−^ mice through its anti-inflammatory [124] and lipid-lowering effects [125], but whether this is due to sirtuin activation is less clear. Nevertheless, resveratrol has many atheroprotective effects on SMC phenotype through SIRT1-dependent mechanisms. For example, resveratrol reduces SMC proliferation and migration evoked by atherogenic stimuli (e.g., oxLDL, high glucose) by inhibition of key pathways like PI3K/Akt/mTOR [126,127]. It also promotes SMC differentiation toward a contractile phenotype through SIRT1- and AMPK-dependent mechanisms [128] and attenuates the osteoblastic transition and calcification in IL-1β-induced senescent SMCs through blocking the NF-κB/p53/p21 pathway [94]. Moreover, resveratrol counteracts AngII-induced SMC senescence by reducing oxidative stress by upregulating the SOD2 antioxidant defense mechanism [129], it upregulates NAMPT in human aortic SMCs [130] and reduces the proinflammatory properties of the SASP of aged SMCs isolated from non-human primates [131]. However, due to its poor bioavailability and low specificity, small-scale trials did not show clear cardiovascular benefit (reviewed in [132]), and so more specific SIRT1 activators with superior pharmacokinetic properties are currently being developed (reviewed in [133]).

#### 5.3.2. Senolytic Drugs

The combination of quercetin (Q), another well-known flavonoid and sirtuin activator, and the tyrosin kinase dasatinib (D) is the most well-studied senolytic cocktail. D (5 mg/kg) + Q (10 mg/kg) increases mouse lifespan [134], alleviates metabolic syndrome [135], and reduces senescence burden and intimal calcification in ApoE^−/−^ mice [79]. Also, quercetin alone (100 mg/kg) reduces plaque size in HFD-fed ApoE^−/−^ mice [136]. While they have not been tested yet in clinical trials specific for atherosclerosis, they have been evaluated in various aging-related conditions. For example, D+Q successfully reduces adipose tissue senescence cell burden in patients with diabetic kidney disease [137], and quercetin is currently being tested as a monotherapy for its anti-senescence and anti-inflammatory effects in patients undergoing coronary bypass surgery (NCT04907253) [138]. Furthermore, there is mechanistic evidence that quercetin has beneficial effects on SMC phenotype and senescence. For example, quercetin induces senolysis of oxidative stress–induced senescent SMCs in an AMPK-dependent manner [139]. Besides suppression of SMC proliferation and migration through MAPK pathways [140], quercetin attenuates the transition of SMCs into macrophage-like foam cells via the JAK2/STAT3/KLF4 pathway in vitro [136], illustrating its potential ability to interfere with the progression of atherosclerosis through inhibition of both SMC senescence and maladaptive SMC phenotypic modulation. Fisetin, another flavonoid with senolytic effects in mouse aorta through acting on the PI3K-Akt-Bcl2 pathway [141], has been shown to reduce atherosclerosis and improve lipid profiles in mice by downregulating PCSK9 and lectin-like oxLDL receptor 1, associated with reduced expression of senescence markers [142]. In vitro, fisetin inhibits SMC senescence by suppressing ROS-NOX1 signaling [143] and reduces SMC calcification by negatively regulating p38 MAPK-dependent pro-calcific signaling [144]. Clinical trials are currently in progress for testing its potential to improve vascular function in the elderly (NCT06133634) and in patients with peripheral artery disease (NCT06399809). Navitoclax (aka ABT-623), which has shown promise in preclinical studies, as discussed in this work, is not currently in clinical trials for CVD, likely because of its severe side effects such as thrombocytopenia [145].

## 6. Future Perspectives

There is compelling evidence that SMC senescence promotes atherosclerotic plaque destabilization by reshaping the plaque composition through inflammatory signaling and modulating SMCs into maladaptive cell states. However, many questions remain unanswered. For example, it is still unclear how many senescent SMCs accumulate in human atherosclerotic plaques at any given disease stage and how they compare to other senescent cells in the plaque such as EC and macrophages. It is also not known whether SMCs from different regions in the aorta have a different susceptibility towards senescence. Given the regional and vascular-bed-specific differences in how SMCs respond to inflammation, lipid exposure, and altered hemodynamics, as well as in their transcriptional profiles [146], it is highly plausible that this translates into a differential propensity to senescence. SMC phenotypic modulation is a reversible process, and the current literature advocates to aim future therapeutic approaches at reversing the unfavorable SMC phenotypes into plaque-stabilizing ones, yet it is unclear if this is still possible once senescence is established. As such, we propose to target SMC senescence instead, either through senolytic or senomorphic agents. However, there are still several concerns about their specificity and safety in humans. Indeed, many senolytics exert toxic side effects and are not specific for senescence, nor for SMCs. The development of anti-senescence strategies is being challenged by the lack of reliable detection methods and the heterogenous characteristics of senescent cells and their SASP [147]. Hence, future studies should be directed at improving the characterization of senescent SMCs by studying their transcriptome, proteome, and secretome to identify targetable biomolecules, for example, those expressed on their surface or released in the blood. Investigation of the transcriptional and epigenetic regulators of SMC senescence as well as their metabolic alterations will further enhance the discovery of cell-specific molecular targets. Overall, the identification of SMC-specific senescence markers will facilitate the assessment of senescence burden in clinical samples and will bring us closer to developing new anti-senescence strategies that specifically target senescent SMCs, or their SASP components, in CVD without harming regenerative cells or causing side effects.

## 7. Conclusions

Cell senescence promotes atherosclerosis and contributes to SMC loss and thinning of the protective fibrous cap. Senescent SMCs largely exert these effects through their pro-inflammatory secretome, yet new evidence shows that they also promote plaque instability by favoring the transition of SMCs into plaque-destabilizing phenotypes. Hence, cell senescence is not merely a terminal cell fate but acts as a critical regulator of SMC plasticity. Additionally, anti-senescence drugs may hold promise as new anti-atherosclerotic therapies to selectively target the pro-inflammatory and plaque-destabilizing SMC phenotypes.

## Data Availability

Not applicable.
